# Supplementary material for: Reconfigurable Metalens with Phase-Change Switching between Beam Acceleration and Rotation for 3D Depth Imaging
Source: Micromachines (Basel). 2022 Apr 13;13(4):607. doi: 10.3390/mi13040607 (PMC9031172; doi:10.3390/mi13040607)
Supplement: Supplementary file 1 [file micromachines-13-00607-s001.zip › micromachines-1638420-supplementary.pdf]

Supplementary Materials

Reconfigurable Metalens with Phase-Change Switching between Beam Acceleration and Rotation for 3D Depth Imaging

Zhiyuan Ma [1,2,3,†], Siyu Dong [1,2,3,*,†], Xiong Dun [1,2,3], Zeyong Wei [1,2,3], Zhanshan Wang [1,2,3,4] and Xinbin Cheng [1,2,3,4]

1 MOE Key Laboratory of Advanced Micro-Structured Materials, Shanghai 200092, China; 1851924@tongji.edu.cn (Z.M.); dunx@tongji.edu.cn (X.D.); weizeyong@tongji.edu.cn (Z.W.); wangzs@tongji.edu.cn (Z.W.); chengxb@tongji.edu.cn (X.C.)
2 Institute of Precision Optical Engineering, School of Physics Science and Engineering, Tongji University, Shanghai 200092, China
3 Shanghai Frontiers Science Center of Digital Optics, Shanghai 200092, China
4 Shanghai Institute of Intelligent Science and Technology, Tongji University, Shanghai 200092, China
* Correspondence: dongsy@tongji.edu.cn
† These authors contributed equally to this work.

Citation: Ma, Z.; Dong, S.; Dun, X.; Wei, Z.; Wang, Z.; Cheng, X. Reconfigurable Metalens with Phase-Change Switching between Beam Acceleration and Rotation for 3D Depth Imaging. *Micromachines* **2022**, *13*, 607. https://doi.org/10.3390/mi13040607

Academic Editors: Benfeng Bai, Yuancheng Fan and Yu-Sheng Lin

Received: 28 February 2022
Accepted: 9 April 2022
Published: 13 April 2022

Publisher's Note: MDPI stays neutral with regard to jurisdictional claims in published maps and institutional affiliations.

Copyright: © 2022 by the authors. Licensee MDPI, Basel, Switzerland. This article is an open access article distributed under the terms and conditions of the Creative Commons Attribution (CC BY) license (https://creativecommons.org/licenses/by/4.0/).

S1: Influence of phase design on the rotation rate of DH-PSF

As supplement of extended depth of field, the DH-PSF metalens designed by us able to generate the focused rotating beam. The phase distribution calculate formula [1] as below:

$$\Phi_{DH}=\frac{2\pi}{\lambda}\left(f-\sqrt{\rho^2+f^2}\right)+\begin{cases}(2n-1)\theta, & \sqrt{\frac{n-1}{N}}<\rho<\sqrt{\frac{n}{N}},\quad n=1,2,\ldots,N\\ 0, & \rho>1\end{cases} \tag{1}$$

This formula means the phase distribution in polar coordinate (ρ,θ), constant N is sum of Fresnelzone. Figure S1 shows the relationship curves between rotation angle and depth corresponding to different values of N. In the design of this paper, we selected $N=4$, the working depth are $3.5\ \text{cm}-13.5\ \text{cm}$. For the other working depth which need be realized, we also can design through change N, bore size of metalens and others. The Figure 3b shown the imitation unified DH-PSF which generated by the DH-PSF metalens when object distance at $3.8\ \text{cm}, 7.6\ \text{cm}, 11.5\ \text{cm}$. From the figure we can clearly see that DH-PSF clockwise revolve along with depth increasing.

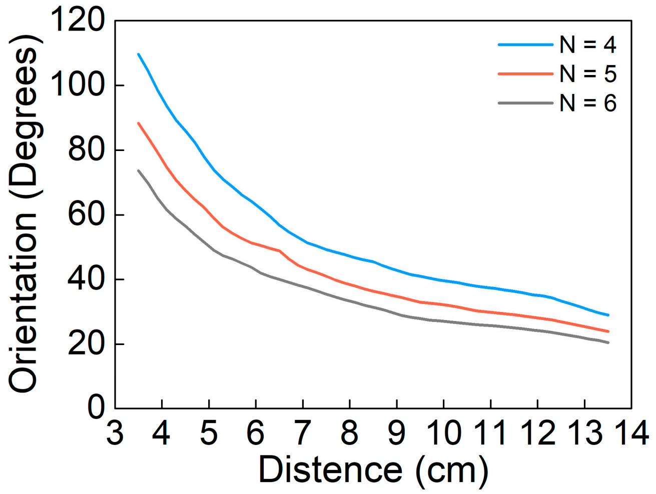

Figure S1. Relation curves of DH-PSF rotation angle and depth corresponding to different N values. When N is set to different values, simulate the relationship curve between the rotation angle and object distance of the designed DH-PSF. With the increase of N, the rotation rate of the designed DH-PSF decreases with the increase of depth.

S2: Geometric dimension design of unit structure

We used the Lumerical FDTD solutions commerce software to imitate unit structure nanorods with different period under TM polarization light normal incidence. The incidence light wave length is $1550\ \mathrm{nm}$. In order to achieve better functional effect of the metalens, we need a sufficient number of unit structures to realize the phase distribution of the metalens, so the period should not be too large. Shown as Figure S2, the magnetism field range magnetic field amplitude $|H|$ in these unit structures under two states when at $p = 0.6\ \mu\mathrm{m} - 0.9\ \mu\mathrm{m}$ period. By observing their magnetic field distribution, we can judge whether the light field is well confined to each nanorods. It can be seen from Figure S2c,d that when the period is large, the light is well limited in the nanorods. This means the partial phase transmission mainly controlled by unit structures under this situation [2,3], almost no coupling between the adjacent unit structure, so we can ignore the affection between the adjacent nanorods [4]. Finally, we chose the period is $0.8\ \mu\mathrm{m}$. Then we can assume that the adjacent nanorods we designed are weakly coupled. And we selected the unit structure size at $100\ \mathrm{nm} - 700\ \mathrm{nm}$. In Figure S3, we drew the curve that phase cover range change along with unit structure height. We chose the height is $1.6\ \mu\mathrm{m}$ under the situation that make ensure the unit structures not coupling, phase of C-state unit structures able to cover 2π range along with that size of a change from $100\ \mathrm{nm}$ to $700\ \mathrm{nm}$. And the phase cover of the GST nanorods has multiplied four-folds

from C-state to A-state.

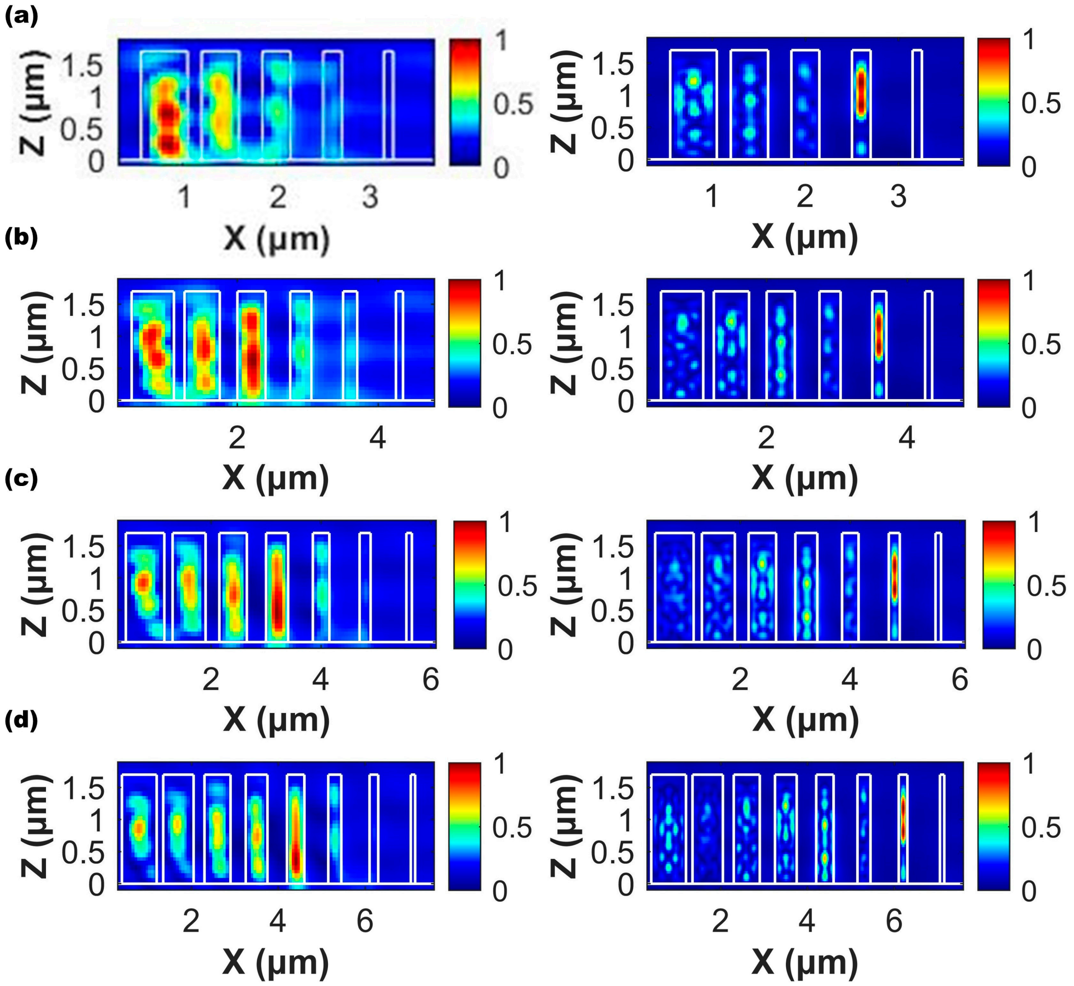

Figure S2: Near field distribution in GST rectangular nanorods of C-state (left) and A-status (right). The height of nanorods is 1.6 μm . (**a**) Period is 0.6 μm , edge length of unit structures from left to right are 0.5 μm, 0.4 μm, ... 0.1 μm respectively. (**b**) Period is 0.7 μm, edge length of unit structures from left to right are 0.6 μm, 0.5 μm, ... 0.1 μm respectively. (**c**) Period is 0.8 μm, edge length of unit structures from left to right are 0.7 μm, 0.6 μm, ... 0.1 μm respectively. (**d**) Period is 0.9 μm, edge length of unit structures from left to right are 0.8 μm, 0.7 μm, ... 0.1 μm respectively.

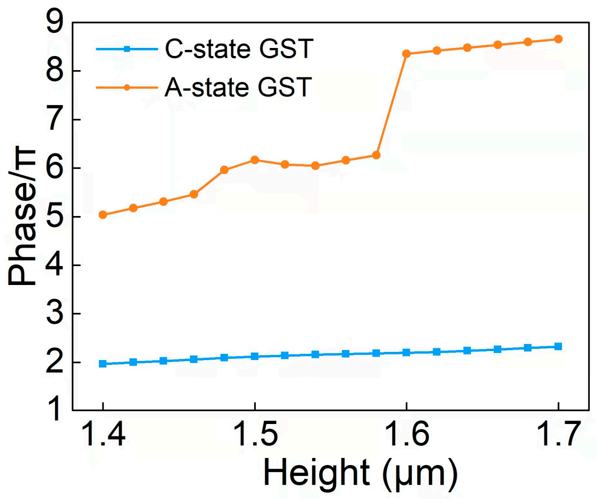

Figure S3: Phase coverage of GST rectangular nanorods in C and A states at different heights. The height of the nanorods is $1.4\ \mu m - 1.7\ \mu m$. Step length $\Delta h = 0.02\ \mu m$, side length size $a = 100\ nm - 700\ nm$.

S3: Design methodology of the metalens

The design of the bifunctional metalens requires each nanorod to meet different phases in the A-state and C-state of GST. Thus, the phase of the metalens in different states is changed to achieve the purpose of a metalens to realize two functions through phase transition. Two phase distribution of metalens are formula (2) and (3) respectively. Here worthy to attention that, though the coordinate (x, y) in phase distribution function is continuous, but the position of unit structure on the metasurface is discrete. So we can only calculate out the required ideal phase value of each one position unit structure on the metasurface. The ideal phase values of the nanorods at (x, y) position on the metalens are calculated as $\varphi_{EDOF(x,y)}$ and $\varphi_{DH(x,y)}$ by formula (2) and (3) in the text. We took the phase scanned by the side length $a = 0.1\ \mu m - 0.7\ \mu m$ parameter of the nanorods in the A-state and C-state as two databases. Each database is a collection of 301 groups (side length a, phase φ). They are $\varphi_A\{a_1, a_2, \ldots, a_n\}$ and $\varphi_C\{a_1, a_2, \ldots, a_n\}$ respectively. Since the quality of reconstructed image using EDOF metalens will also affect the acquisition of DH-PSF and depth estimation of DH-PSF metalens, we need to give priority to ensuring the function of EDOF metalens. Finally, we designed EDOF metalens in A-state and DH-PSF metalens in C-state. Because the metalens needs to match the two ideal phases of the nanorods in two states at each (x, y) position [5]. So, it existing deviation between the actual phase $\varphi\{a_r\}$ and ideal phase of unit structure, they are $\Delta\varphi_A = |\varphi_A\{a_r\} - \varphi_{EDOF(x,y)}|$ and $\Delta\varphi_C = |\varphi_C\{a_r\} - \varphi_{DH(x,y)}|$ respectively. When designing

adjustable metasurfaces, some artificial intelligence algorithms can be used to screen the elements. However, because the bifunctional metalens designed in this paper contains 1250×1250 unit structures and each unit is a variable, using the traditional artificial intelligence algorithm requires a lot of iterations and a large amount of calculation. Therefore, when determining the side length dimension a of the unit structure, we minimized the total error $\Delta\varphi = \Delta\varphi_A + \Delta\varphi_C$ at (x, y) position, so as to select the r dimension value a_r in the database. This method able to make ensure that metalens phase match with ideal phase at the same time of reduce large quantity calculation. Repeat the above method of select the unit structure size till finish fill the whole metalens.

S4: Overall size distribution of the metalens

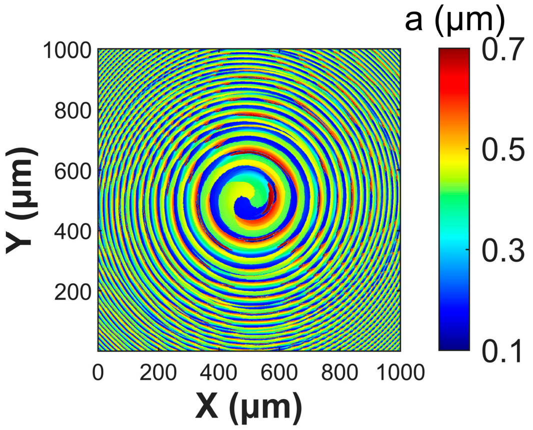

Figure S4: The dimensions of all unit structures of the designed metalens. The side length of the metalens is 1 mm, the period of the unit structure is 0.8 μm, and there are 1250×1250 unit structures in total. We use different colors to represent different side length values a.

S5: Polarization insensitive property

Due to the centrosymmetry of the nanorods we select, the metalens we designed is polarization insensitive [3]. In Figure S5, we drew the electric field distribution of a row of unit structures (ten nanorods in Figure 2b) in the $X - Y$ plane when TM and TE polarized light are incident respectively. It can be seen from the figure that the response of the unit structure to incident light with different polarization is basically the same.

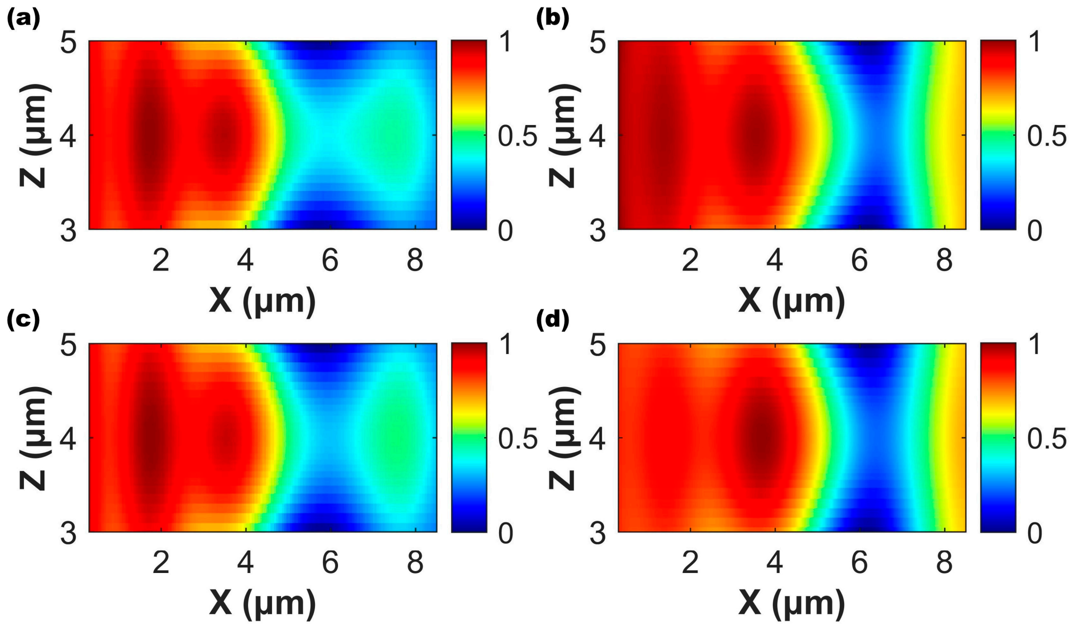

Figure S5: Electric field distribution of a row of unit structures (ten nanorods in Figure 2b) in the X-Y plane with TM and TE polarized light. (**a**) Electric field distribution of C-state GST nanorods with TM polarized light. (**b**) Electric field distribution of A-state GST nanorods with TM polarized light. (**c**) Electric field distribution of C-state GST nanorods with TE polarized light. (**d**) Electric field distribution of A-state GST nanorods with TE polarized light. The wavelength of incident light is 1550 nm. The direction of incident light is the same as that in Figure 2b, which is incident in the normal direction along the Z axis. The field distribution monitor is placed 10 μm below the ten unit structures.

S6: Uniformity of different depth EDOF-PSFs

Aim to the metalens actually imitated PSF (Figure 3a), we used MATLAB software to verify that EDOF metalens generated EDOF-PSF at different depth are basically not changed. We select the EDOF-PSF with a depth of 7.0 cm as the calibration PSF, and compare the similarity between the three EDOF-PSFs (Figure 3a) simulated and the calibration PSF. Shown as the below Table S1, R is the relate coefficient which calculated by MATLAB. The closer R is to 1, the greater the similarity between the two images. On the contrary, the closer R is to 0, the smaller the similarity. From Table S1 we can see that, the similar among three EDOP-PSF all achieved that $R > 0.9$, so we can ensure that EDOF-PSF almost not changed along with depth change.

Table S1: Similarity calculation of EDOF-PSF of EDOF metalens which generated at three different depths[1]

Serial number	Depth[2]	R[3]
1	3.8	0.9037
2	7.6	0.9992
3	11.5	0.9860

[1] Calculate the similarity between EDOF-PSF at three different depths and EDOF-PSF at 7.0 cm.

[2] The depth is the distance between the point source and the metalens.

[3] R is correlation coefficient. The similarity calculation uses the corr2 function in MATLAB to solve the 2-D correlation coefficient R between the two matrices. The range of R is 0 to 1.

The metalens we designed has a resolution of 625 lp/mm. Taking the depth of 9.5 cm as an example, at this depth, we calculated that the Strehl ratio of EDOF metalens was 0.401. Here, we also referred to the reference [5] and used MTF to evaluate EDOF metalens. Figure S6 shows the MTF of the EDOF metalens with actual phase at different depths (3.8 cm, 7.6 cm, 11.5 cm). It can be seen that the designed metalens has a highly depth-invariant modulation transfer function compared to a design without the wavefront coding term added, which means it can produce depth insensitive EDOF-PSF, so as to reconstruct clear images.

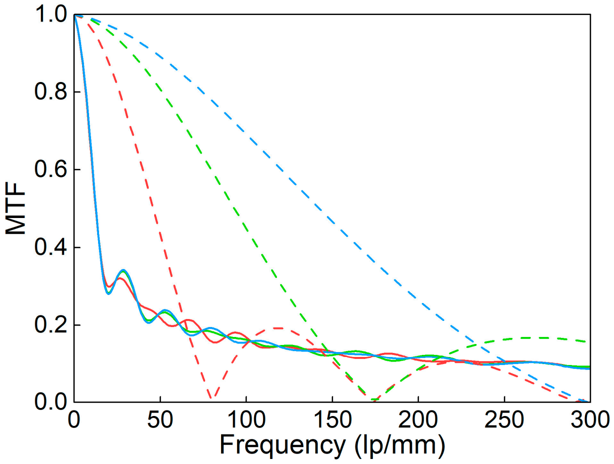

Figure S6: Modulation transfer function of EDOF metalens and standard metalens. Compared with the modulation transfer function of standard metalens (dotted lines) without cubic phase term, EDOF metalens (solid lines) we designed has a highly depth-invariant MTF. The distances of point light sources are 3.8 cm (red), 7.6 cm (green) and 11.5 cm (blue).

S7： Method of 3D scene reconstruction

The object light with different depth is received by the detector after passing through the metalens, and then we reconstruct a clear object depth image through the received blurred image. (1) Obtain the vague images on the detector: we used MATLAB software to imitate the

generated vague image that detector observed incidence light with object information passed through the metasurface. Firstly, we imitated the actual PSF of metalens at different depth. Then folding the object light and imitate PSF through MATLAB software then got the vague image after object passed through the metasurface. For the object light, we all added the noise with variance 0.02. (2) Reconstruct clear images: Due to the strong invariability of EDOF-PSF with depth, in the process of scene reconstruction at different depths, our PSF can select EDOF-PSF at any working depth as the fixed template PSF. Here we used EDOF-PSF at fix depth 9.5 cm. Further more, we used the scene reconstruction algorithm based on regularization filter algorithm to deconvolute the blurred image generated by EDOF metalens with the above fixed EDOF-PSF to reconstruct the clear image. (3) Depth estimation: After reconstructing the image, we used the depth estimation algorithm based on Wiener filter algorithm to deconvolute the blurred image generated by DH-PSF metalens with the clear image just reconstructed. Then we can get the DH-PSF of the object at this depth. For dual objects scene, we selected the sub area of each interesting object through cutting images. We processed depth estimation at each one sub area respectively. In the process of calculating the angle of DH-PSF, we ignored some uninterested regions outside the two focal points, such as sidelobe and noise. For the two main lobes of DH-PSF, we calculated their centroids and connect them, so as to calculate its rotation angle. Finally, we created the clear depth image through that estimated depths combine with the reconstructed clear images.

Table S2 and Table S3 indicated the theory value, estimation value and error of single object and dual objects under different depth respectively.

Table S2: Theory value, estimation value and error of five different depths in single object depth imaging

Theoretical depth[1] (cm)	DH-PSF rotation angle[2] (°)	Estimated depth[3] (cm)	Relative error[4]
3.7	104.405	3.701	0.03%
5	76.4324	4.965	0.70%
7.4	50.7145	7.225	2.36%
9.6	39.8057	9.928	3.42%
12.7	34.5024	12.266	3.41%

[1] Theoretical depths are five different depths for a single object we set.

[2] We use the depth estimation algorithm to obtain the estimated rotation angle of DH-PSF for

each object.

[3] Estimated depths are the depths estimated through the rotation angle of DH-PSF and the theoretical curve (Figure 4c).

[4] Relative error represents the difference between the estimated depth and the theoretical depth.

Table S3: Theory value, estimation value and error of two different depths in dual objects depth imaging

Theoretical depth[1] (cm)	DH-PSF rotation angle[2] (°)	Estimated depth[3] (cm)	Relative error
4.2	90.1288	4.262	1.48%
10.4	37.4465	10.986	5.63%

[1] The theoretical depths are the depths of each of the two objects we set.

[2] We use the depth estimation algorithm to obtain the estimated rotation angle of DH-PSF for each object.

[3] Estimated depths are the depths estimated by simulating the whole physical process.

From Figure 3c, we can see that the DH-PSF metalens we designed can realize DH-PSF which is very sensitive to depth change. Its rotation angle can correspond to the depth one by one. Therefore, it can be well used to estimate the depth. For the estimation accuracy of DH-PSF metalens in crystalline state, we can see the specific error values in Table S2 and Table S3 above. It can be seen that DH-PSF metalens has 3.42% error in single object depth imaging and 5.63% error in dual objects depth imaging.

References

1. Wang, Z.J.; Cai, Y.N.; Liang, Y.S.; Dan, D.; Yao, B.L.; Lei, M. Aberration correction method based on double-helix point spread function. *J. Biomed. Opt*. **2019**, *24*, 031005.
2. Hu, Y.Q.; Wang, X.D.; Luo, X.H.; Ou, X.N.; Li, L.; Chen, Y.Q.; Yang, P.; Wang, S.; Duan, H.G. All-dielectric metasurfaces for polarization manipulation: Principles and emerging applications. *Nanophotonics* **2020**, *9*, 3755–3780.
3. Ou, K.; Li, G.; Li, T.; Yang, H.; Yu, F.; Chen, J.; Zhao, Z.; Cao, G.; Chen, X.; Lu, W. High efficiency focusing vortex generation and detection with polarization-insensitive dielectric metasurfaces. *Nanoscale* **2018**, *10*, 19154–19161.
4. Wang, S.; Wu, P.C.; Su, V.-C.; Lai, Y.-C.; Chen, M.-K.; Kuo, H.Y.; Chen, B.H.; Chen, Y.H.; Huang, T.-T.; Wang, J.-H.; et al. A broadband achromatic metalens in the visible. *Nat. Nanotechnol.* **2018**, *13*, 227–232.
5. Shalaginov, M. Y.; An, S.; Zhang, Y.; Yang, F.; Su, P.; Liberman, V.; Chou, J. B.; Roberts, C. M.; Kang, M.; Rios, C.; Du, Q.; Fowler, C.; Agarwal, A.; Richardson, K. A.; Rivero-Baleine, C.; Zhang, H.; Hu, J.; Gu, T. Reconfigurable all-dielectric metalens with diffraction-limited performance. *Nat. Commun.* **2021**, *12*, 1225.
6. Colburn, S.; Majumdar, A. Metasurface Generation of Paired Accelerating and Rotating Optical Beams for Passive Ranging and Scene Reconstruction. *ACS Photonics* **2020**, *7*, 1529–1536.
